# Hepatitis B Virus Genotype G: The Odd Cousin of the Family

**DOI:** 10.3389/fmicb.2022.872766

**Published:** 2022-03-31

**Authors:** Natalia M. Araujo, Carla Osiowy

**Affiliations:** ^1^Laboratory of Molecular Virology, Oswaldo Cruz Institute, FIOCRUZ, Rio de Janeiro, Brazil; ^2^National Microbiology Laboratory, Public Health Agency of Canada, Winnipeg, MB, Canada

**Keywords:** hepatitis B virus, genotype G, HBV/G, HIV co-infection, MSM, phylogeography

## Abstract

With a widespread distribution but low prevalence worldwide, the hepatitis B virus (HBV) genotype G (HBV/G) is a recently described genotype for which the origin and biology are poorly understood. Some unique features make HBV/G the most peculiar of all genotypes. In this review, we reflect on the major milestones in HBV/G research, highlighting the main aspects of its discovery, molecular epidemiology, and virological and clinical characteristics. We also illustrate common pitfalls in the routine detection, which may lead to underestimated rates of HBV/G infection. Large-scale analysis of data from dozens of articles was further performed, with the aim of gaining comprehensive insights into the epidemiological aspects of HBV/G. Finally, we point out recent findings on HBV/G origins and discuss new perspectives regarding the evolutionary history of HBV/G and the plausibility of an African geographic re-emergence of this genotype.

## Introduction

Hepatitis B is one of the most prevalent viral infections in humans and a major global public health problem. Hepatitis B virus (HBV) has infected one-third of the global population, with an estimated 296 million chronic infections, and more than 800,000 deaths in 2019, mostly from cirrhosis and hepatocellular carcinoma (HCC). Despite the availability of a prophylactic vaccine, 1.5 million new infections are reported each year (WHO, 2021). New therapeutic agents for treatment of HBV are urgently needed, as currently available treatments, such as pegylated-interferon alpha and third-generation nucleos(t)ide analogue therapies, result in low functional cure (HBsAg clearance) rates; thus, long-term treatment adherence is required to effectively control HBV replication (Alter et al., 2018; Revill et al., 2019). HBV is transmitted through contact with bodily fluids, mainly in the context of sexual and perinatal transmission and exposure to infected blood. Due to shared modes of transmission, co-infection with HIV is common, with an estimated 2.7 million people living with both infections (Singh et al., 2017).

Even though HBV has been infecting humans for millennia, there is little detailed knowledge of how the disease spread among populations and geographical areas in the past. Remarkably, recent findings described the discovery and sequencing of HBV genomes using archeological remains up to 10,500 years old from Eurasia and the Americas (Kocher et al., 2021) to describe the timing and pathway of HBV evolution in humans. HBV is the prototype member of the family *Hepadnaviridae*, containing a partially double-stranded relaxed circular DNA genome approximately 3,200 nucleotides (nt) in length (Seeger et al., 2013). This family is characterized by a unique viral replication cycle that involves the activity of an error-prone reverse transcriptase, generating the emergence of significant diversity among HBV isolates (Osiowy et al., 2006; Revill et al., 2020). Based on >7.5% genomic sequence divergence, HBV has been phylogenetically classified into nine genotypes (A–I; Kay and Zoulim, 2007; Kramvis, 2014), with a putative genotype (J) isolated from a single individual (Tatematsu et al., 2009). The significant diversity within specific HBV genotypes has led to further classification into numerous subgenotypes (Shi et al., 2013; Pourkarim et al., 2014). Since HBV has no known environmental or animal reservoirs, its spread is therefore tightly linked to the dispersal of humans. The global distribution of HBV genotypes displays a significant geographic association, which most likely reflects historic patterns of human migrations (Zehender et al., 2014; Paraskevis et al., 2015). Moreover, frequencies of clinically relevant mutations (i.e., immune escape-, stop codon-, drug resistance-, and HCC-associated mutations) vary among HBV genotypes (Araujo et al., 2020), while differences in transmission routes, disease progression, response to antiviral therapies, and clinical outcome have also been demonstrated (Chu and Lok, 2002; Lin and Kao, 2011).

In this review, we explore the major milestones in research on HBV genotype G (HBV/G), a rare, recently described genotype for which the biology is poorly understood. We provide an overview from its discovery, molecular epidemiology, and virological and clinical characteristics to recent findings on HBV/G origins. Data from dozens of articles were also collected with the aim of gaining comprehensive insights into the epidemiological and biological aspects of HBV/G, usually referred to as the “odd,” “aberrant,” and “mysterious” HBV genotype.

## Genotype G

### Discovery of a Genotype With Unique Characteristics

HBV/G was first described in 1990 as an HBV variant detected in an HIV-infected homosexual man from San Francisco, California (Bhat et al., 1990). One year later, a similar variant was described from a French HBV chronic carrier (Tran et al., 1991). However, the formal report of HBV/G as a new human HBV genotype was only published in 2000, when this genotype was found in chronically infected patients from Atlanta (Georgia, United States) and Lyon (France), and a complete genome sequence was described (Stuyver et al., 2000).

Some unique characteristics make HBV/G the most peculiar of all the HBV genotypes. It has a genomic length of 3,248 nt, making it longer than isolates of the other HBV genotypes, which range from 3,182 to 3,221 nt. The longer length of the HBV/G genome is attributed to a 36-nt insertion located after the fifth nucleotide following the core translation initiation point (A at position 1901), which leads to a 12 amino acid (aa) insertion at the amino-terminal end of the core protein (HBcAg; Stuyver et al., 2000). Other remarkable features of HBV/G are two translational stop codons at aa positions 2 and 28 of the pre-core coding region that prevents the expression of the hepatitis B e antigen (HBeAg), which appears to be essential for the establishment of a persistent HBV infection (Kato et al., 2002a; Hadziyannis, 2011). This likely explains why in chronic infections HBV/G is invariably found to co-infect with another HBV genotype capable of furnishing HBeAg *in trans* (Sugiyama et al., 2007; Sakamoto et al., 2013). Similar insertions and stop codons have been exclusively found in ancient HBV genomes (dating back 7,000 years) from which modern HBV/G descends (Kocher et al., 2021). The 36-nt insertion is absent in other HBV genotypes most likely because it results in downregulation of genome replication through diminished polymerase expression (Gutelius et al., 2011). Furthermore, the 36-nt insertion has been shown to result in decreased transcription of the pregenomic RNA replicative transcript (Li et al., 2007), likely due to modifications in the core promoter or enhancer II regulatory regions, despite the presence of multiple promoter mutations enhancing replication (Gutelius et al., 2011). In contrast, the 36-nt insertion appears to regulate and increase core protein expression (Gutelius et al., 2011; Basic et al., 2021), upon which HBV/G is dependent for replication (Li et al., 2007), to ensure adequate capsid assembly in the context of reduced replication. The 12-aa insertion has been shown to result in enhanced genome maturation (Li et al., 2007), possibly due to reduced efficiency of capsid envelopment or due to unique HBV/G amino acid substitutions (Cotelesage et al., 2011; Gutelius et al., 2011). Moreover, HBV/G patients are often immunocompromised due to HIV co-infection, which tolerates the high expression of core protein, without stimulating immune clearance mechanisms (Zhang et al., 2019). Additionally, the HBV/G genome has a 2-aa deletion in the HBcAg carboxy-terminal region (as with all other HBV genotypes except HBV/A) and a 1-aa deletion in the pre-S1 region (shared with HBV/E; Stuyver et al., 2000). At the nucleotide level, the intra-genotype divergence among HBV/G isolates is remarkably low (<0.7%), while other HBV genotypes demonstrate divergence values up to 6%. However, the inter-genotype divergence displayed by HBV/G at >11.3% is significantly higher than the minimum threshold of >7.5% distinguishing different HBV genotypes (Kato et al., 2002b). Curiously, HBV/G has a 30-nt segment in the pre-S1 region that is almost identical to HBV/E but which differs substantially from the other genotypes (Lindh, 2005), as discussed further below.

### Clinical Associations With HBV/G Infection

The prospect of HBV/G being directly cytopathic has been suggested by observations of cellular HBcAg accumulation or HBsAg retention in an immune-deficient mouse model or *in vitro* culture, respectively (Sugiyama et al., 2007; Peiffer et al., 2015; Hassemer et al., 2017). The high levels of HBV-DNA often observed during HBV/G co-infection are a further hallmark associated with the advancement of liver disease (Dao et al., 2011). Indeed, we have previously showed that HBV/G has by far the highest frequency (≥95%, *p* < 0.001) of mutations associated with an increased risk for HCC (C1653T, T1753V, and A1762T/G1764A) in comparison with other genotypes (Araujo et al., 2020). However, the role of HBV/G in HCC development remains to be established. Despite the difficulty in differentiating the clinical outcome contribution of HBV/G and its co-infecting genotype, a number of studies have described a significant association of increased liver fibrosis with HBV/G infection in the context of HIV co-infection (Lacombe et al., 2006; Dao et al., 2011; Adachi et al., 2016). In multivariate analysis among 158 prospectively followed HIV-HBV-co-infected patients, HBV/G was found to be independently associated with fibrosis progression (*p* < 0.01) as determined by FibroTest (Malagnino et al., 2019). Importantly, the rate of progression was most pronounced in patients having low-baseline METAVIR scores (F0–F2). However, fibrosis was not found to be associated with HBV/G co-infection in HIV-infected patients studied retrospectively (Calin et al., 2013). Differences among studies may be due to the level of immunosuppression among study participants, comorbidities or other co-infections, and the co-infecting HBV genotype.

Although HBV/G appears to respond to HBV antiviral therapies in a similar fashion as other genotypes (Baldick et al., 2008; Erhardt et al., 2009), a higher frequency (32.5%, *p* < 0.001) of mutations resulting in resistance to lamivudine (strains carrying reverse transcriptase M204V/I) was observed in this genotype (Araujo et al., 2020). In addition, a reduction in susceptibility to tenofovir disoproxil fumarate (TDF), a third-generation nucleotide analogue, has been suggested. In particular, clinical follow-up among HIV-HBV-co-infected individuals treated with TDF following non-response to lamivudine primary treatment showed a delayed response (median 20 months) to TDF, primarily among HBV/G compared to other genotype infections (*p* = 0.026; Lada et al., 2012a). Interestingly, *in vitro* cell culture using HBV/G replicative constructs also displayed a diminished susceptibility to TDF-treated cells (Lada et al., 2012b).

### A Ubiquitous but (Apparently) Minority Genotype

**Figure 1:**
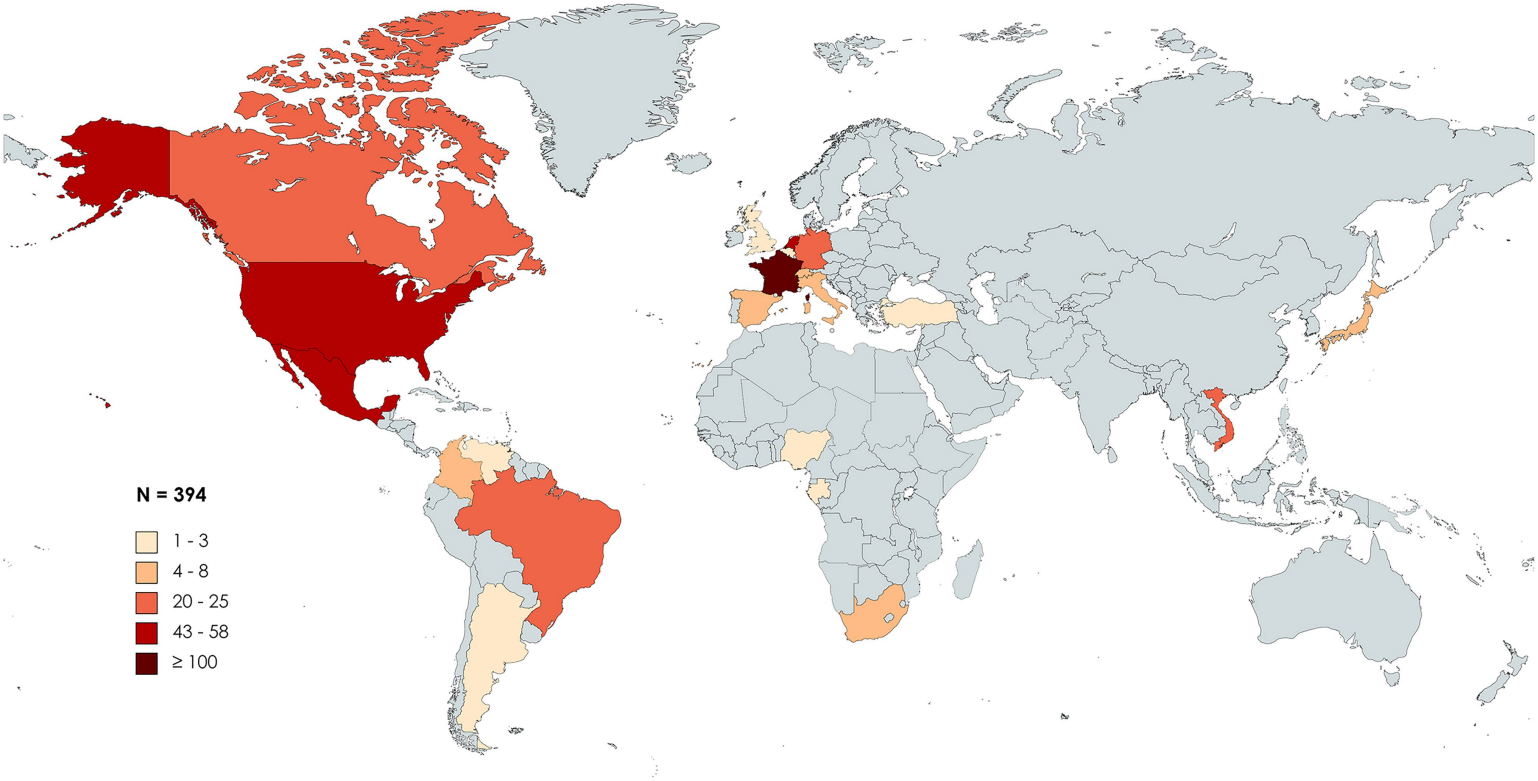
Geographic distribution of genotype G (HBV/G)-infected patients. Global map distribution based on absolute number of HBV/G-infected patients in each country extracted from 55 articles analyzed in this study. The map was created with mapchart.net.

First discovered in patients from France and the United States, HBV/G was later found among all five continents. Following a PubMed MEDLINE database search [“(hepatitis B virus) AND (“genotype G”)”], 55 studies reporting cases of HBV/G infection were collected and analyzed (Table 1). Studies involving recombinants without detection of HBV/G non-recombinant strains were not included in the analysis. At present, HBV/G infection has been described in Argentina, Brazil, Canada, Colombia, Mexico, United States, and Venezuela (the Americas); Belgium, France, Germany, Italy, Netherlands, Spain, Switzerland, Turkey, and the United Kingdom (Europe); Gabon, Nigeria, and South Africa (Africa); Japan and Vietnam (Asia; Figure 1). In absolute figures, the highest number of patients infected with HBV/G has been found in Europe (*n* = 204), followed by the United States (*n* = 152), Asia (*n* = 29), and Africa (*n* = 9). By country, France detected the highest number of HBV/G-infected patients (*n* = 101; Figure 1; Table 1). However, in terms of proportion of infection, two studies from Mexico reported the highest rates (40% and 43%, respectively) of HBV/G in HIV-co-infected patients (Mata Marin et al., 2012; Jose-Abrego et al., 2021; Table 1). Of note, the high prevalence of HBV/G among the Mexican population has been discussed elsewhere (Roman and Panduro, 2013).

**Table 1:** HBV/G-infected patient data from 55 literature references.

References	Country	No. of HBV/G-infected patients (%)	Genotype in co-infection	Main patient group	Age	Gender
Araujo et al., 2013	Argentina	1	F	HIV+	NI	1 M
	Brazil	1	F	HIV+ and MSM	NI	1 M
Pourkarim et al., 2011	Belgium	2 (4.3)	NI	HIV+	NI	2 M
Bottecchia et al., 2008	Brazil	3 (8.3)	NI	HIV+ and MSM	44–51	3 M
de Barros et al., 2015	Brazil	1	A2	NI	32	1 M
Lampe et al., 2017	Brazil	13 (1.3)	NI	NI	NI	NI
Silva et al., 2010	Brazil	2 (13)	A	HIV+ and MSM	NI	2 M
Osiowy et al., 2015	Canada	12 (0.7)	NI	NI	NI	NI
Osiowy et al., 2008	Canada	13 (1.8)	A	MSM	25–70	11 M/1 F
Alvarado Mora et al., 2011	Colombia	4 (7.7)	NI	Blood donor	37–56	4 M
Calin et al., 2013	France	31 (24.8)	NI	HIV+ and MSM	38–46	21 M/2 F
Lacombe et al., 2006	France	25 (12.1)	NI	HIV+	40.8 (mean)	13 M
Tran et al., 1991	France	1	NI	NI	NI	NI
Malagnino et al., 2019	France	25 (15.8)	A	HIV+	35–40	25 M
Lada et al., 2012a	France	9 (23.7)	A	HIV+	Adults	Mostly M
Fallot et al., 2012	France	8 (24.2)	A, D, F	HIV+	NI	NI
Stuyver et al., 2000	France	2 (5.1)	NI	NI	NI	NI
	United States	11 (13.4)	NI	NI	NI	NI
Toan et al., 2006	Gabon	2 (4.3)	A, C, D	NI	Children	NI
	Germany	7 (18.4)	A, C, D	NI	Adults	NI
	Vietnam	19 (5.1)	A, C, D, F	HIV−	Adults	NI
Vieth et al., 2002	Germany	1	NI	HIV+	Adult	1 M
Basic et al., 2021	Germany	12 (2.1)	A, E, D	HIV−	Adults	NI
Chudy et al., 2006	Germany	1	Mono-infection	Blood donor	60	1 M
Zehender et al., 2003	Italy	4 (12.1)	NI	HIV+	NI	NI
De Maddalena et al., 2007	Italy	4 (4)	NI	HIV+	NI	NI
Tamada et al., 2012	Japan	1 (0.2)	A	HIV−	Adult	NI
Shibayama et al., 2005	Japan	1 (2.4)	A2	HIV+ and MSM	40	1 M
Kato et al., 2004b	Japan	0 (0)	NA	NI	NA	NA
Kojima et al., 2013	Japan	3 (20)	NI	HIV+	NI	NI
Jose-Abrego et al., 2021	Mexico	10 (40)	A2, D, H	HIV+	Adults	NI
Sanchez et al., 2002	Mexico	1 (6.7)	NI	Sexual worker	Adult	1 F
Fernandez-Galindo et al., 2015	Mexico	2 (9.1)	NI	HIV+	33–47	2 M
Mata Marin et al., 2012	Mexico	16 (43.2)	NI	HIV+	NI	NI
		0 (0)	NI	HIV−	NI	NI
Escobedo-Melendez et al., 2014	Mexico	2 (8.3)	NI	Blood recipient	Children	NI
Alvarado-Esquivel et al., 2006	Mexico	5 (10.2)	NI	HIV+	NI	NI
Sanchez et al., 2007	Mexico	7 (28)	A, H	MSM	NI	Mostly M
		0 (0)	NA	Non-MSM	NA	NA
Cotten et al., 2014	Netherlands	1	NI	HIV+	Adult	NI
Cornelissen et al., 2016	Netherlands	45 (19.9)	A	HIV+ and MSM	NI	NI
van der Kuyl et al., 2013	Netherlands	10	A2	HIV+	NI	10 M
Lieshout-Krikke et al., 2014	Netherlands	1	Mono-infection	Blood donor	42	1 M
Zaaijer et al., 2011	Netherlands	1	Mono-infection	Blood donor	NI	1 M
Forbi et al., 2010	Nigeria	3 (12.5)	E	NI	19–23	2 M/1 F
Lukhwareni et al., 2020	South Africa	1 (3.3)	NI	HIV+	Adult	NI
Smuts et al., 2017	South Africa	3 (1.4)	A	HIV+ and MSM	31–48	3 M
Jardi et al., 2008	Spain	6 (5.8)	A, D	HIV−	Adults	Mostly M
Rodriguez-Frias et al., 2006	Spain	1 (1.3)	A	NI	NI	NI
Hirzel et al., 2015	Switzerland	5 (1.1)	A	NI	NI	NI
Sayan and Dogan, 2012	Turkey	1	A	Hemodialysis	61	1 M
Ganova-Raeva et al., 2015	United Kingdom	1	A2	HIV−	62	1 M
Bhat et al., 1990	United States	1	NI	HIV+ and MSM	43	1 M
Dao et al., 2011	United States	18 (13.5)	NI	HIV+	24–52	17 M/1 F
Chu et al., 2003	United States	6 (1.1)	NI	NI	Adults	NI
Teshale et al., 2011	United States	8 (1.3)	NI	NI	NI	NI
Thai et al., 2012	United States	2 (18.2)	A	NI	38–44	2 M
Kato et al., 2002a	United States	4	A	NI	27–41	4 M
Kato et al., 2004a	United States	8 (4.8)	A	NI	38.4 (mean)	6 M/2F
Jaspe et al., 2014	Venezuela	1 (3.6)	Mono-infection	HIV+ and MSM	41	1 M
Hoan et al., 2021	Vietnam	5 (2.4)	NI	NI	Adults	NI

*NA, not applicable; NI, not informed; M, male; and F, female*.

Despite its wide geographic distribution, the global prevalence of HBV/G seems to be low. In fact, studies that assessed the frequency of HBV/G infection in the general population of HBV carriers (without focusing on any risk group) have reported very low rates of HBV/G, ranging from 0% to 1.3% (Chu et al., 2003; Kato et al., 2004b; Teshale et al., 2011; Tamada et al., 2012; Hirzel et al., 2015; Osiowy et al., 2015; Lampe et al., 2017; Table 1). However, it is very likely that HBV/G is underreported. In the vast majority of published cases, HBV/G is detected in co-infection with another HBV genotype isolate, often as the minor variant among the viral quasispecies population. Single time-point, cross-sectional analysis of patient samples may not allow the detection of HBV/G during co-infection, due to fluctuating viral load levels, and thus, detection will be dependent on the genotyping assay limit of detection. It is noteworthy that routine PCR amplification of the polymerase-coding region has been reported to be unsuccessful to detect HBV/G in European inactive carriers, while HBV/G-specific primers and deep sequencing determined HBV/G to form the major quasispecies in HBV/A-co-infected carriers (Basic et al., 2021). Thus, HBV genotyping assays employing deep sequencing methodologies or amplification with HBV/G-specific or highly sensitive universal primers may overcome the presumed lack of detection of this genotype, and so likely modify its true worldwide prevalence.

### Genotype G-Infected Patients and Major Risk Factors

Data extracted from a portion of the 55 articles described transmission risk factors associated with HBV/G infection. Eleven articles (20%) noted HBV/G infection in men who have sex with men (MSM), with a further 26 articles (47%) observing HBV/G-HIV co-infection (Table 1). In particular, a previous study analyzing the HBV genotype distribution in two groups of MSM and non-MSM Mexican patients found HBV/G infection exclusively in MSM patients (4/25, 28% MSM; 0/42 non-MSM; Sanchez et al., 2007). Another study from Mexico detected HBV/G infection in 43.2% (16/37) of HIV-co-infected patients and in none (0/40) of the HBV mono-infected patients (Mata Marin et al., 2012). Also, in HIV-co-infected patients from France, a 25% (31/125) HBV/G prevalence was found, while none (0/98) of the HBV mono-infected patients was infected with this genotype (Desire et al., 2011). Thus, since unprotected anal sex is an important risk factor for HIV infection, it seems to be a major risk factor for the acquisition of HBV/G. In addition, the prevalence of HBV/G infection was similar between HIV-positive and HIV-negative MSM patients from the Netherlands (Cornelissen et al., 2016), suggesting that present HIV infection apparently does not act as an additional risk factor. Of note, only in two studies (2/55, 4%) has HBV/G been detected in children (two from Gabon and two from Mexico; Toan et al., 2006; Escobedo-Melendez et al., 2014), which indicates that perinatal (mother-to-infant by blood exposure at the time of birth) and early childhood (familial- and community-based) transmissions are not major routes for HBV/G infection. Also, according to gender data extracted from the 55 articles in Table 1, HBV/G infection is significantly more common in men (139 males and 8 females).

A total of 4 out of the 55 (7%) articles described HBV/G infection in blood donors; 3 of them (3/4, 75%) confirming HBV/G as the only HBV genotype (Chudy et al., 2006; Alvarado Mora et al., 2011; Zaaijer et al., 2011; Lieshout-Krikke et al., 2014; Table 1). These three cases of HBV/G mono-infection occurred in HBsAg-negative subjects, which hampers its detection by serological diagnosis. These results demonstrate that routine serology may be unsuitable for the detection of HBV/G mono-infection, and thus, data on its occurrence may be underestimated.

### Co-infection With Other HBV Genotypes

In 25 (25/55, 45%) articles, information on the HBV/G-co-infecting genotype was described (Table 1). The following genotypes were detected: A (23/25, 92%), D (5/25, 20%), F (3/25, 12%), H (2/25, 8%), E (2/25, 8%), and C (1/25, 4%). Triple genotype mixtures, such as G/A/D, G/D/E, and G/D/H, were detected in patients with HIV and were significantly associated with higher HBV viral loads and liver fibrosis (Fallot et al., 2012; Jose-Abrego et al., 2021). The rare reports of HBV/G co-infection with HBV/C and HBV/E, as well as the absence of data of co-infection with HBV/B, may be explained by the low prevalence of HBV/G in Asia and Africa, where these genotypes are endemic (C and B in Asia, E in Africa; Kay and Zoulim, 2007; Kramvis, 2014). Therefore, it seems that the co-infecting genotype is normally that which is highly prevalent within the geographical region, suggesting that HBV/G can associate with any other HBV genotype capable of supplying HBeAg. As differences among HBV genotypes exist regarding the timing of HBeAg seroconversion (Livingston et al., 2007) and prevalence of pre-core or basal core promoter mutations that result in loss or reduced HBeAg (Rodriguez-Frias et al., 2006; Ito et al., 2018), HBV/G co-infection with genotypes demonstrating stable HBeAg expression should be an evolutionary advantage. For example, HBV/C, which exhibits delayed seroconversion (Livingston et al., 2007), has been described in recombination with HBV/G (Suwannakarn et al., 2005; Toan et al., 2006; Tangkijvanich et al., 2013). Similarly, subgenotypes A1 and A3, in contrast to subgenotype A2, have been described to undergo early HBeAg seroconversion (Ingasia et al., 2021), with A2 most frequently associated with HBV/G co-infection as detailed below. Curiously, HBV/I (a complex intergenotypic recombinant of genotypes A, C, and G) has been frequently identified in China and India, although G has never been described as a parent genotype in these countries (Fang et al., 2011; Haldipur et al., 2014).

The very high rates of HBV/G co-infection observed with HBV/A (92%) are very interesting and lead to certain speculations. When information is available regarding the HBV/A subgenotype, A2 is consistently reported (Table 1), which may be associated with the geographic location of these studies (North America and Europe) where subgenotype A2 is much more prevalent than A1 (Ito et al., 2018). In the host dynamic context of HBV as a communicable disease, a certain density and mobility of infected and naïve hosts are requisite for ongoing sexual transmission; thus, natural selection tends to favor long-term persistence (chronicity) and reduced lethality of sexually transmitted pathogens (Ewald, 2004). Subgenotype A2 seems to best meet these conditions, mainly due to its longer duration of HBV viremia and mild acute hepatitis in infected patients (Araujo et al., 2011; Ito et al., 2018). Of note, subgenotype A2 appears to be spreading within the MSM community in Japan, where the national prevalence of A2 is low (Fujisaki et al., 2011). Remarkably, a recent study has demonstrated that HBcAg expression from HBV/G is favored when this genotype is co-expressed with HBV/A (and E to a lesser extent), favoring HBV/G dominant encapsidation and virus assembly, but not when it is co-expressed with HBV/D (Basic et al., 2021). Therefore, all these findings suggest that co-infection among HBV/G and A2 may occur preferably, which might explain the propensity for sexual transmission of HBV/G.

Trans-complementation and other studies have shown that co-infecting HBV genotypes further contribute to HBV/G infection aside from the contribution of HBeAg toward the development of chronic infection. *In vitro* and chimeric mouse studies have shown that HBV/G mono-infection results in very low viral replication, likely due to reduced levels of transcribed pregenomic RNA during replication (Sugiyama et al., 2007; Gutelius et al., 2011; Sakamoto et al., 2013). Pregenomic RNA serves as the template for both HBV replication and translation of the viral core and polymerase proteins, and due to a series of mutations in the HBV/G basal core promoter and enhancer II element, reduced pregenomic RNA transcription and polymerase translation is thought to contribute to the reduced viral replication observed (Gutelius et al., 2011). Co-infection with another HBV genotype rescues this defect in part by increasing HBV/G replication through upregulation of HBV/G core translation, although this effect is pleiotropic depending on the co-infecting genotype (Sugiyama et al., 2007; Gutelius et al., 2011; Sakamoto et al., 2013). Co-infection also modulates HBV/G HBsAg expression (Sakamoto et al., 2013), although the expressed protein localizes to the perinuclear region likely due to the HBV/G-specific pre-S1 sequence resulting in misfolded protein (Hassemer et al., 2017; Basic et al., 2021). The cellular retention of HBV/G HBsAg results in ER stress (Peiffer et al., 2015) and secretion defects, which are rescued during co-infection with HBV/A (Sakamoto et al., 2013).

During HBV/G replication, virion particles were observed to have more complete relaxed circular DNA, indicating greater virion maturation prior to envelopment (Dryden et al., 2006; Gutelius et al., 2011). This observation may be due to reduced particle secretion as a result of inefficient capsid development and envelopment of HBV/G particles (Li et al., 2007). HBV ultrastructural studies have shown the 12-aa insertion at the HBV/G core protein N-terminus to form a mass at the base of capsid protein dimers, which likely sterically hinders the interaction of several essential residues required for contact with the cytoplasmic domain of HBsAg, leading to inefficient envelopment (Cotelesage et al., 2011). Furthermore, the amino acid mass partially covers residues within the hydrophobic pocket, which undergo structural changes following envelopment, and thus are associated with virion secretion. Hindrance of virion envelopment and secretion permits increased genome maturation, which is in keeping with the HBV/G core amino acid mass not interfering with the diffusive movement of nucleotide triphosphates into the capsid interior (Cotelesage et al., 2011). Sakamoto et al. (2013) have speculated that core protein from the co-infecting “helper” genotype may replace HBV/G core during virion packaging in natural infection to compensate for these defects and allow for increased envelopment and secretion efficiency.

### Ancient but Unknown Origin

HBV/G has been described in different and distant countries worldwide, making its geographic origin unclear. A possible African geographic origin of HBV/G has been hypothesized (Lindh, 2005), based on its similarity with HBV/E, which is endemic in Africa (Kramvis and Kew, 2007; Ingasia et al., 2021). Both genotypes have a very low degree of genetic diversity and each forms a single monophyletic group with no subgenotypes, suggesting a recent evolutionary history in humans. HBV/G is the least divergent from HBV/E (11%), sharing with this genotype an almost identical 30-nt segment in the pre-S1 region, which differs substantially from other genotypes (Lindh, 2005). According to Lindh’s hypothesis, HBV/G may have acquired the 30-nt fragment through a recombination event with HBV/E. Since HBV/E is rare outside West and Central Africa, HBV/G may have originated within this region (Lindh, 2005). The fact that HBV/G is frequently found in co-infection with HIV, which has been proposed to originate from West/Central Africa (Sharp and Hahn, 2011), corroborates this hypothesis. In addition, previous reports of HBV/G co-infection with HBV/E in individuals of African ethnicity, including those from remote communities in rural Nigeria (Forbi et al., 2010; Basic et al., 2021), give some strength to this hypothesis. Interestingly, both studies showed that HBV/G was frequently found as the minor variant in HBV/E co-infection, which may lead to underestimated rates of G/E co-infection, dependent on the genotype testing method. On the other hand, the high prevalence of HBV/G and HBV/H co-infection reported in Mexico is puzzling, which leads to the plausibility of the hypothesis that both HBV/G and HBV/H are endemic to Mexico (Roman and Panduro, 2013).

**Figure 2:**
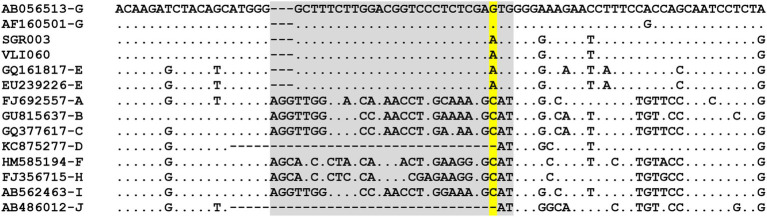
Comparison of partial pre-S1 sequences from HBV/A to HBV/J and two ancient DNA sequences related to HBV/G (SGR003 and VLI060, published in Kocher et al., 2021). Region where HBV/E and HBV/G share a unique sequence (shaded in gray). Nucleotide site where HBV/G differs from its ancestral sequences and HBV/E (shaded in yellow). HBV reference sequences based on McNaughton et al. (2020).

Just recently, Kocher et al. (2021) have identified 14 ancient HBV genomes from skeletal remains (dating between ~7 and 3.5 thousand years ago) that carry insertions and stop codons similar to present-day HBV/G strains and forming a subclade from which modern HBV/G descends. These ancestral strains prevailed throughout western Eurasia for ~4,000 years, declining around the end of the 2nd millennium BCE. HBV/G is the only lineage remnant of this apparently now-extinct prehistoric clade that appears to have re-emerged. Based on ancient DNA studies, HBV/G seems to be the most phylogenetically distant of the modern human HBV genotypes (Kocher et al., 2021). The remarkably low genetic diversity found among current HBV/G strains suggests viral persistence and a recent re-emergence from the ancient lineage, as opposed to a recent evolutionary introduction of HBV/G. This reappearance is thought to have occurred in association with the HIV pandemic, after thousands of years of low-level persistence (Kocher et al., 2021). Indeed, phylodynamic patterns have pointed to a sharp increase of HBV/G dissemination co-occurring with the HIV pandemic at the beginning of the 1990s, possibly associated with highly sexually active groups and injection-drug users (Wolf et al., 2021). However, speculation regarding the geographic and temporal origins of re-emerged HBV/G is tempered by the differing timescales (i.e., ancient vs. contemporary DNA sequences) used in phylogenetic analyses, which will confound the time to the most recent common ancestor. The original HBV/G genomic signal was present in a distinct “Western Eurasian Neolithic to Bronze Age” clade based on the phylogeographical analyses of Kocher et al. (2021) incorporating ancient DNA. It is likely that spread to West Africa and South/Central America are important components of this origin story, as HBV/E and F/H share extant sequence signatures common to HBV/G that are also found in ancient DNA, within the pre-S1 region (specific to HBV/E, nt 2,853–2,879; numbering based on accession no. GQ161817, HBV/E) and the core protein sequence (specific to HBV/E, F, H, aa179P, and 180A; numbering based on accession no. AY090461, HBV/F; Gutelius et al., 2011). Remarkably, the 30-nt sequence fragment shared amongst HBV/G and HBV/E is found in two HBV/G ancestral genomes dating from the Bronze Age (SGR003 and VLI060; Kocher et al., 2021). The sequence fragment is identical to the HBV/E fingerprint, differing by one nucleotide (A2880G, numbering based on accession no. GQ377617, HBV/C) in modern HBV/G (Figure 2). Although A2880G is well conserved in HBV/G (virtually all HBV/G sequences in accessible databases carry this mutation), it is likely incidental, since A2880G results in a synonymous amino acid change in pre-S1 (Glu in both HBV/G and E) and a non-synonymous, but equivalently nonpolar amino acid change, in the polymerase spacer domain (Met in HBV/E and Val in HBV/G), thus not seeming to confer an evolutionary advantage for HBV/G. Altogether, these findings allow us to propose an update of Lindh’s hypothesis presented 17 years ago, suggesting that the re-emergence (and not emergence) of HBV/G might have occurred in West/Central Africa, with HBV/G as the donor of the 30-nt fragment to HBV/E (and not the other way around) by recombination, probably at a time prior to the rise of A2880G in HBV/G.

## Conclusion

In conclusion, HBV/G has unique molecular and epidemiological characteristics, which make it the most peculiar of all the HBV genotypes. Despite its widespread geographic distribution, HBV/G has been little reported worldwide. However, fluctuating viral load levels of HBV/G during co-infection with another genotype, as well as the hampered production of HBsAg in HBV/G mono-infection, may lead to undetectable HBV/G and, consequently, an underreported prevalence of this genotype. The association of increased liver fibrosis with HBV/G infection in HIV-co-infected individuals, and the possible reduction in susceptibility to TDF, primarily observed in HIV and HBV/G co-infection, highlight the importance of HBV genotyping assays employing deep sequencing methodologies or amplification with HBV/G-specific or highly sensitive universal primers in the context of HIV co-infection. MSM appears to be a population at major risk for HBV/G infection and transmission. From an evolutionary perspective, the very high rates of HBV/G-HBV/A2 co-infection *via* sexual transmission may have preferentially arisen as the latter genotype is more likely to be HBeAg-positive and cause mild acute hepatitis in infected patients. The recent discoveries of HBV/G ancestral strains that prevailed throughout western Eurasia for ~4,000 years shed light on new perspectives regarding the origins of HBV/G. The remarkable finding of sequence signatures shared amongst modern HBV/G and HBV/E strains in ancient genomes most closely related to HBV/G merges the evolutionary histories of these two genotypes and strengthens the idea of an African geographic re-emergence of HBV/G and posterior global spread co-occurring with the HIV pandemic.

## Author Contributions

NMA and CO designed the study, searched and collected the literature, wrote, revised, and finalized the manuscript. All authors contributed to the article and approved the submitted version.

## Funding

NMA: Conselho Nacional de Desenvolvimento Científico e Tecnológico (CNPq), grant number 428676/2018-9, and Fundação de Amparo à Pesquisa do Estado do Rio de Janeiro (FAPERJ), grant number E-26/210.450/2019.

## Conflict of Interest

The authors declare that the research was conducted in the absence of any commercial or financial relationships that could be construed as a potential conflict of interest.

## Publisher’s Note

All claims expressed in this article are solely those of the authors and do not necessarily represent those of their affiliated organizations, or those of the publisher, the editors and the reviewers. Any product that may be evaluated in this article, or claim that may be made by its manufacturer, is not guaranteed or endorsed by the publisher.
